# Effects of graphene on morphology, microstructure and transcriptomic profiling of *Pinus tabuliformis* Carr. roots

**DOI:** 10.1371/journal.pone.0253812

**Published:** 2021-07-08

**Authors:** Xiao Zhang, Huifen Cao, Haiyan Wang, Runxuan Zhang, Haikuan Jia, Jingting Huang, Jianguo Zhao, Jianzhong Yao

**Affiliations:** 1 Key Laboratory of National Forest and Grass Administration for the Application of Graphene in Forestry, Institute of Carbon Materials Science, Shanxi Datong University, Datong, P.R. China; 2 College of Life Science, Shanxi Datong University, Datong, Shanxi Province, PR China; 3 College of Chemistry and Chemical Engineering, Shanxi Datong University, Datong, P.R. China; 4 National Fine Variety Base of Pinus sylvestris var. in Honghuaerji Forestry Bureau, Hulunbeir Inner Mongolia, PR China; 5 Shanxi Poplar High-yield Forest Bureau, Datong, Shanxi Province, PR China; University of Naples Federico II, ITALY

## Abstract

Graphene has shown great potential for improving growth of many plants, but its effect on woody plants remains essentially unstudied. In this work, *Pinus tabuliformis* Carr. bare-rooted seedlings grown outdoors in pots were irrigated with a graphene solution over a concentration range of 0–50 mg/L for six months. Graphene was found to stimulate root growth, with a maximal effect at 25 mg/L. We then investigated root microstructure and carried out transcript profiling of root materials treated with 0 and 25 mg/L graphene. Graphene treatment resulted in plasma-wall separation and destruction of membrane integrity in root cells. More than 50 thousand of differentially expressed genes (DEGs) were obtained by RNA sequencing, among which 6477 could be annotated using other plant databases. The GO enrichment analysis and KEGG pathway analysis of the annotated DEGs indicated that abiotic stress responses, which resemble salt stress, were induced by graphene treatment in roots, while responses to biotic stimuli were inhibited. Numerous metabolic processes and hormone signal transduction pathways were altered by the treatment. The growth promotion effects of graphene may be mediated by encouraging proline synthesis, and suppression of the expression of the auxin response gene *SMALL AUXIN UP-REGULATED RNA 41* (*SAUR41*), *PYL* genes which encode ABA receptors, and *GSK3* homologs.

## Introduction

Graphene is an important carbon nanomaterial with unique physical and chemical properties, such as a colossal surface area, robust thermal and electrical conductivity, and good mechanical strength, which make it a chosen material for nanoelectronics [1], biomedicine [2], mechanical engineering [3], and environmental governance [4]. It is estimated that at least 1.3 billion dollars will be injected to develop new applications for graphene from 2014 to 2024 [5]. With the growing use of graphene materials in more and more fields, there is an increasing public concern about the safety of their releases to the environment [6]. Hence, research on the biological effect of graphene has become an emerging topic in recent years.

It is generally believed that the effect of graphene on plant growth is associated with its physiochemical properties, the application method used, the level and duration of exposure, and the plant species being examined [7]. Numerous studies have reported positive effects of graphene on the growth of wheat [8], coriander [9], garlic plants [9], maize [10], spinach [11], chive [11], *Gossypium hirsutum* [12], *Catharanthus roseus* [12], Arabidopsis [13], watermelon [13] and *Aloe vera* [14] at concentrations ranging from 10–200 mg/L. Graphene could promote plant growth through several different mechanisms. As a water transporter, graphene could accelerate water absorption in roots and seeds [11]. Graphene treatment may enhance ROS scavenging capacity to alleviate oxidative stress, enhance soluble protein content, and decrease cell death [10]. Moreover, the application of graphene may improve fertilizer utilization efficiency and affect the activity of soil microorganisms and soil organisms, thus indirectly affecting plant growth [6, 15–17].

Notwithstanding these positive effects, it is reported that graphene may be detrimental to plants under certain conditions. The sharp edges of graphene may physically cut cell membranes and compromise their integrity [18]. In addition to increasing the uptake of water and fertilizer by roots, graphene also increased the uptake of heavy metals such as cadmium and arsenic, which increased their toxic effects [19, 20]. Furthermore, graphene treatment may lead to the alteration of pH, metabolic processes, induce different degrees of oxidative damage, and cause cell death [21]. These reported negative effects underscore the necessity for further research before graphene can be applied in agroforestry.

Exogenous stimuli can change plant gene expression profiles, and so provide important indicators for their effects. Graphene oxide treatment changes the transcription level of auxin and abscisic acid synthesis genes in *Brassica napus* L. [22]. The transcript levels of auxin signaling pathway genes *IAAs* and *ARFs* were affected by graphene oxide treatment in tobacco [23]. RNA-seq has been used as an efficient and fast means to discover the effects of graphene treatment. Chen et al. reported that graphene treatment of maize leads to the upregulation of genes related to transcriptional factor regulation, plant hormone signal transduction, nitrogen and potassium metabolism, as well as secondary metabolism, thus providing numerous candidate genes for the graphene response [24].

To our knowledge, previous studies on the biological effects of graphene have focused on herbaceous plants, and woody plants, which have distinct physiological structures and growth processes, have not been well investigated. Because of the advantages of strong adaptability, drought resistance, cold resistance, and ability to grow on barren land, *Pinus tabulaeformis*, one of the most widely distributed and important afforestation tree species in northern China, has played an important role in conserving soil and water and improving the environment [25]. Here, *Pinus tabulaeformis* was used to study the events occurring in woody plants in response to graphene treatment using physiological experiments, cytological observations and RNA sequencing. Our study enriches the understanding of the biological effects of graphene and provides a theoretical basis for the application of graphene in agroforestry.

## Materials and methods

### Materials and characterization

All the chemicals and reagents used in this study were analytically pure. Graphene suspension was produced by our laboratory as previously described [24]. In order to verify the quality, the graphene used in this paper was further characterized by scanning electron microscopy and Raman spectroscopy. To obtain the morphology, graphene suspension was vacuum freeze-dried and observed with scanning electron micro-scope (SEM, TESCAN, MAIA 3 LMH). A drop of graphene solution was wind-dried on concave slide for detection of Raman spectra using Renishaw inVia™ Qontor with a 532 nm excitation laser.

### Treatments and plant growth status analysis

A graphene suspension with a concentration of 5 g/L was diluted in ultrapure water to different concentrations (0, 12.5, 25 and 50 mg/L). Two years old pot-grown *P*. *tabulaeformis* bare-rooted seedlings of similar size were selected for experiments. There were 20 ± 3 plants in each group, among which 10 plants were selected for morphological and cytological analysis, and the remaining were used for RNA sample. Every plant was irrigated with 150 mL graphene solution once a month for 6 months.

The fresh weight (FW) of the root tissue was determined and then root morphology analysis was conducted using a root scanner (EPSON Expression; China). The root tissue was then dried at 65°C for 48h and dry weight (DW) was determined [26]. The water content (WC) was calculated as WC = (FW-DW) / FW%. The root scanning images were analyzed by WinRHIZO software (Regent Instrument Inc., Montreal, Canada). Root length (RL), root projected area (RP), root superficial area (RS), root volume (RV), root tip number (RT), and root fork number (RF) were measured to evaluate the root growth status.

### Transmission electron microscopy

Roots were washed in water and placed into 10 mL centrifuge tubes containing 2.5% (v/v) glutaraldehyde dissolved with 50 mM phosphate buffer (pH = 7.2) for primary fixation. The following sample processing and preparation experiments were entrusted to the Electron Microscope and Mass Spectrometry Analysis Platform of the Institute of Food Science and Technology, Chinese Academy of Agricultural Sciences. Images were obtained using a transmission electron microscope (TEM, HITACH, H-7500; Japan). The average root cell area was determined using Image J software to measure the two outermost cell areas in the transmission electron microscope images.

### RNA extraction, library construction, sequencing and bioinformatics analysis

Three biological replicates were performed for both the control (CK) and graphene treatment groups. Cleaned root tissue was wrapped in aluminum foil and immediately immersed in liquid nitrogen. The following experiments, including RNA extraction, cDNA purification, library construction, sequencing and bioinformatics analysis were commissioned to OE Biotech Co., Ltd. (Shanghai, China). The sequencing was conducted using an Illumina HiSeq X Ten sequencer. Raw data were processed with Trimmomatic software to obtain clean reads [27], which were assembled into transcripts by the paired-end method using Trinity software [28]. The raw data of RNA-seq could be obtained from the Genome Sequence Archive in the BIG Data Center of Sciences (https://bigd.big.ac.cn/) under accession number CRA004280. The longest transcripts were chosen as unigenes for subsequent analysis. A final unigene set was obtained by clustering with CD-HIT software [29] to remove redundancy. The methods for functional annotation and classification were described in (S1 File). SwissProt annotation and Gene ontology (GO) classification were performed for the unigene set. The unigenes were mapped to the Kyoto Encyclopedia of Genes and Genomes (KEGG) database to assign them to potential metabolic pathways. The expression abundance of each unigene in each sample was determined by sequence alignments. The Bowtie2 software [30] was used to obtain the number of reads for each unigene in each sample, and the software eXpress was used to calculate the expression of the unigenes according to the FPKM (Fragments Per KB Per Million Reads) method [31]. Genes with a p value < 0.01 and FC (Fold of change) ≥ 2 were considered differentially expressed genes (DEGs), which were identified using DESeq software [32]. DEGs were further annotated using NR and SwissProt annotations from other plant databases. GO enrichment analysis of DEGs was conducted according to the Fisher algorithm, and the KEGG database was used for pathway analysis.

### Quantitative real-time PCR analysis

Total RNA was extracted from CK and 25 mg/L graphene treated groups with the RNAprep pure plant kit (TIANGEN, Shanghai, China), digested with DNase Ⅰ and reversed transcribed into cDNA using PrimeScript^®^ RT reagent Kit (Takara, Dalian, China). The expression of *TUBB* (TRINITY_DN15602_c0_g1_i1_1), which is predicted to encode the tubulin beta-5 chain, was used as an internal control. Primers were designed using the NCBI (National Center for Biotechnology Information) Primer-BLAST program (https://www.ncbi.nlm.nih.gov/tools/primer-blast/) and are listed in S1 Table in S1 File. Quantitative real-time PCR was conducted as described in [33], and the relative level of each gene was calculated using the ΔΔCT (cycle threshold) method. Three independent biological replicates were performed for qRT–PCR.

### Statistical analysis

Each experiment had three biological replicates, and the results are presented as mean ± standard deviation (SD). Significant differences between means were analyzed mainly by one-way analysis of variance using the SPSS 21 software. Significance differences were determined by a least significant difference (LSD) test at a 0.05 probability level.

## Results and discussion

### Characterization of graphene

**Fig 1:**
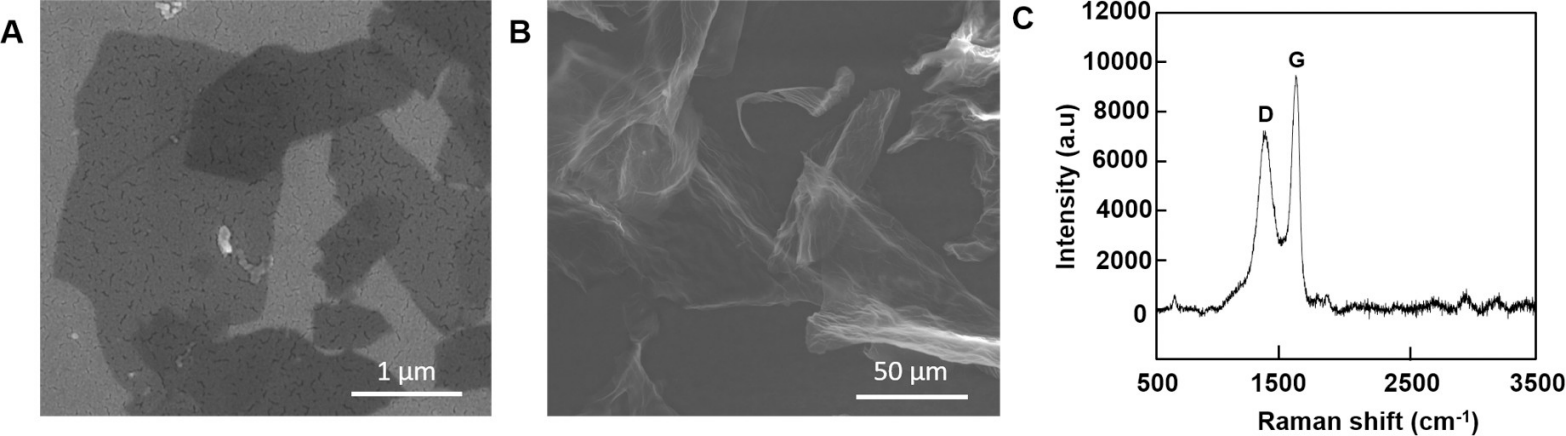
Characterization of graphene. (A, B) Scanning electron microscope images of graphene; (C) Raman spectra of graphene.

Scanning electron microscopy was used to determine the morphological characteristics of the graphene used in this study. At low power, the graphene presented a uniformly distributed lamellar structure, with a scale less than 1.70 × 2.77 μm (Fig 1A). At high power, the graphene displayed a smooth, folded and undulating shape as a whole (Fig 1B). The Raman spectrum of graphene is presented in Fig 1C. D band (~ 1,343 cm−1) and G band (~ 1,559 cm−1), the two main representative Raman peaks of graphene were clearly evident, and the ratio of D band to G band intensity (ID/IG) was about 0.76. These results indicate that the graphene used for this study is of high quality.

### Graphene treatment promote root growth

**Fig 2:**
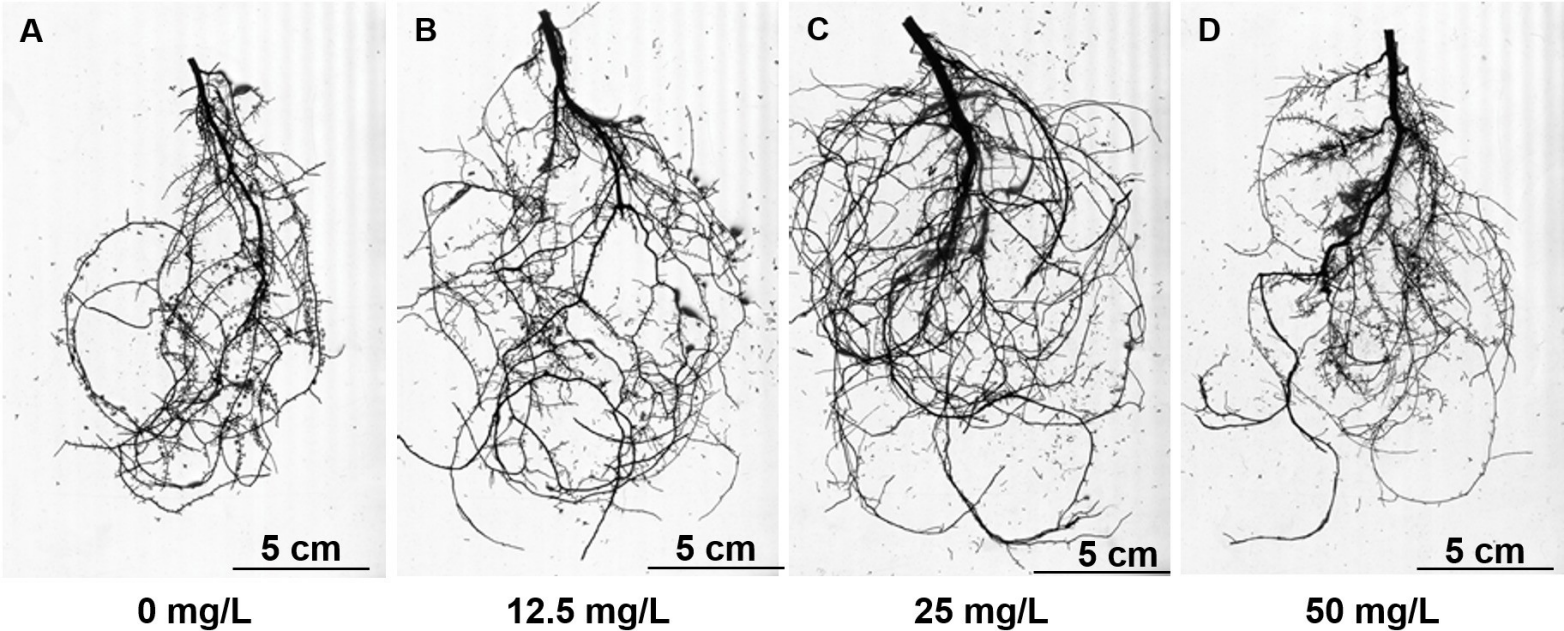
Root morphology of graphene treated roots.

To explore potential applications of graphene in forestry and to study the effects of graphene on woody plants in soil, we studied *P*. *tabulaeformis*. Graphene solutions ranging from 0–50 mg/L were used to irrigate *P*. *tabulaeformis* bare-rooted seedlings once a month. No visible differences were seen in the aerial parts between treatment and CK groups after 6 months (S1 Fig). In contrast to the aerial part, the root of *P*. *tabulaeformis* was more sensitive to graphene treatment (Figs 2 and S1). Compared with the control group, with graphene concentrations ranging from 12.5 to 50 mg/L, the root fresh weight increased by 67.2% to 109.8% (S2 Table in S1 File). Correspondingly, root dry weight of these three groups increased by 65.3% to 103.7% (S2 Table in S1 File). As expected, since the fresh weight and dry weight of the plant roots changed almost in equal proportions among treatment groups, no differences in water content were observed among groups (S2 Table in S1 File).

To further quantify the effect of graphene treatment on *P*. *tabulaeformis* root growth, the roots of different groups were scanned and analyzed. A summary of root morphological parameters, including root length, root projected area, root surface area, root volume, root tip number, and root fork number are shown in S3 Table in S1 File. In accordance with its effects on root biomass, graphene significantly increased almost all root morphology parameters at the tested concentrations. Graphene at a concentration of 25 mg/L increased the root length, root projected area, root surface area, root volume, root tip number and root fork number by 45.6%, 229.8%, 68.0%, 93.8%, 238. 7%, 39.7% and 24.9%, respectively. Based on the results of root biomass and root morphological parameters, the optimal concentration for promoting *P*. *tabulaeformis* root growth in the range of 0–50 mg/L was 25 mg/L, which was used for subsequent analysis.

### Cytological and morphological changes were induced by graphene treatment

**Fig 3:**
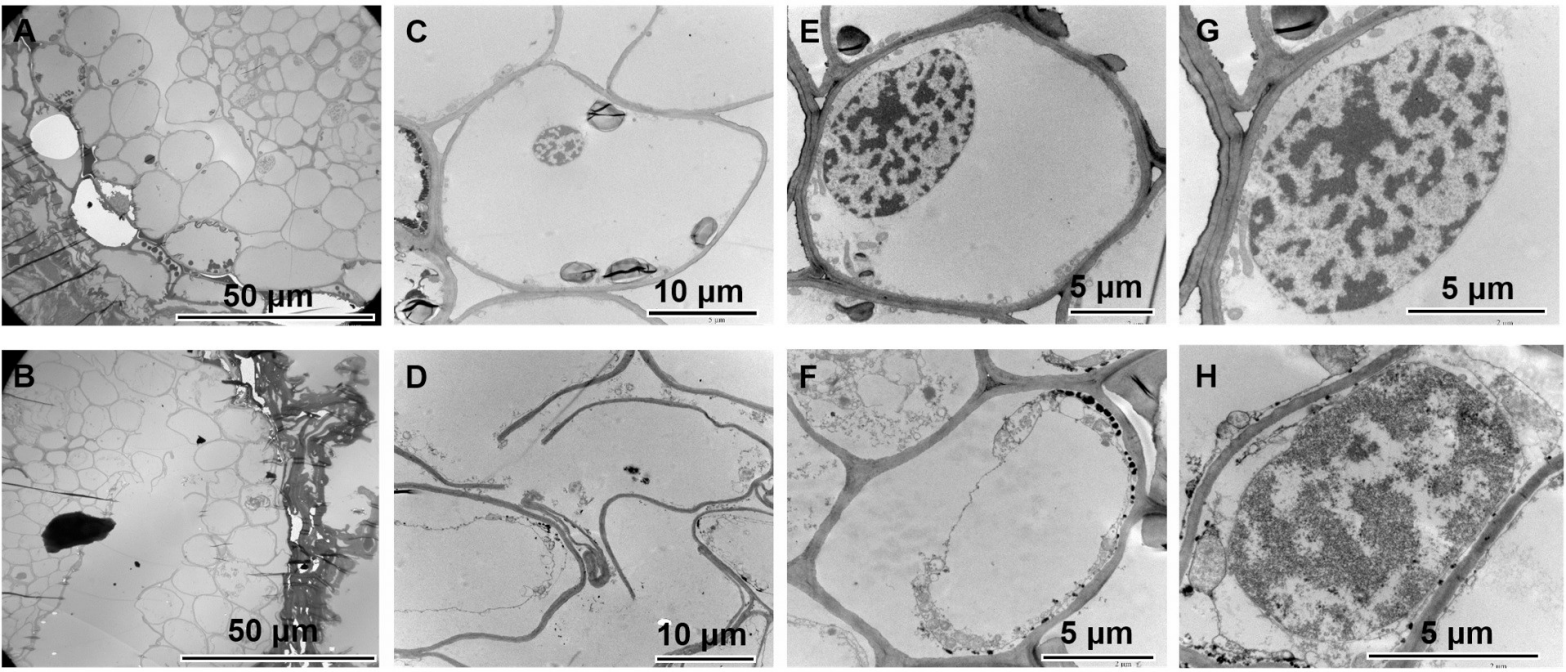
Transmission electron microscopy images of the *P*. *tabulaeformis* root system grown in the absence of graphene (A, C, E and G), and with graphene treatment (B, D, F and H).

To determine whether graphene could be absorbed into the roots of woody plants and to investigate the effect of graphene on root cell structure, the cell morphology of *P*. *tabulaeformis* grown with and without 25 mg/L graphene was observed by transmission electron microscopy. Although graphene particles have previously been observed inside cells by TEM [20], no evidence of this was found in the current study (Fig 3B, 3D, 3F and 3H). We believe that graphene in the soil either cannot enter the roots of woody plants, or that the amount of graphene entering the roots is below the observable threshold.

Studies on herbaceous plants have shown that graphene treatment can cause damage to the cell membrane [34, 35]. The transmission electron microscopy images showed clear evidence of damage to the cell structure in the graphene treatment group relative to the CK (Fig 3). Images of root cross-sections of the control group showed a regular arrangement of cells, with occasional small gaps between cells (Fig 3A). In the graphene treated roots, the gap between cells was significantly enlarged (Fig 3B). High magnification images showed that the cells in the control group were structurally intact (Fig 3C and 3E), while some cells in the graphene-treated group had broken cell membranes (Fig 3D). On the other hand, the graphene treatment did not affect nuclear morphology (Fig 3G and 3H). These results indicate that graphene treatment may cause damage to the plasma membrane, and thus act as a stress.

The size of plant organs is determined by the number and size of cells they contain, which is controlled by cell division and cell expansion, respectively [33, 36]. From the TEM image, we found that the average cell area of the outermost two layers of the plant root decreased by 32.1%, from 525.15 μm^2^ to 356.72 μm^2^ (Fig 3A and 3B). This suggests that the increase of root biomass of *P*. *tabulaeformis* resulting from graphene treatment may be due to an increase in cell number rather than cell volume; graphene treatment may promote cell division in the root system.

### Sequencing and transcriptome assembling

To explore the global transcriptome changes of *P*. *tabulaeformis* in response to graphene treatment, root tissue from 0 and 25 mg/L graphene treated plants were collected and RNA sequencing was carried out with three replicates, with each replicate consisting of 3 randomly selected plants. A total of 320 million raw reads and 317 million clean reads were generated for 6 root cDNA libraries with 94.9% to 96.5% valid bases. The GC content of the reads was between 46.4% and 46.5% (S4 Table in S1 File). A final set of 123462 unigenes were obtained by *de novo* assembly and was used as reference genome sequence for subsequent analysis. The clean reads of each group were mapped to these unigenes. The number of total mapped clean reads was 43.40–50.18 million (86.2% - 89.2%) and the number of uniquely mapped clean reads was 33.72–40.06 million (69.0% - 71.2%) (S4 Table in S1 File). And the results of functional annotation and classification were present in (S1 File and S2 Fig).

### Differentially expressed gene analysis

The expression levels of unigenes were calculated by fragments per kilobase of transcript per million fragments mapped (FPKM) method and normalized using DESeq software. The significance threshold for differential expression was set as |log2(FoldChange)| > 1 and *p*-value < 0.05. In total, 50104 DEGs were observed after graphene treatment in root tissue, among which 21179 were upregulated and 28925 were downregulated. Since most of the annotated genes were homologous to fungal genomes, we removed these and obtained 6477 plant DEGs (S1 Table), of which 2427 were up-regulated and 4050 were down-regulated. The genes matched sequences mainly from the genome of *Arabidopsis thaliana* (55.44%), *Picea sitchensis* (28.39%), *Oryza sativa* (8.74%), *A*. *trichopoda* (2.01%), *Nicotiana tabacum* (1.59%) and *N*. *glutinosa* (1.51%).

### Gene enrichment analysis for DEGs after graphene treatments

**Fig 4:**
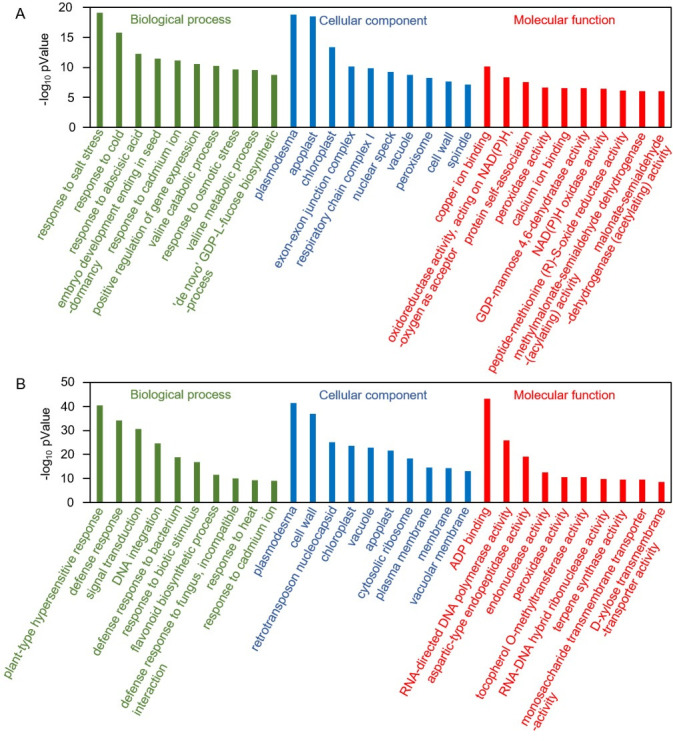
TOP10 GO terms for enrichment analysis of DEGs annotated to plant databases in each of the three GO categories. A: up-regulated DEGs; B: down-regulated DEGs.

To understand the effects of graphene on the *P*. *tabulaeformis* root system, GO enrichment analysis were conducted with DEGs annotated from plant databases. A total of 4871 DEGs, including 1919 up-regulated and 2952 down-regulated, were aligned and classified using the GO database. Fig 4 shows the 10 terms with the highest enrichment degree for 3 categories. After exposed to graphene, GO terms associated with important biological processes were enriched in ‘response to exogenous abiotic and biotic stimulis’. The up-regulated DEGs were mainly enriched for terms related to stress responses, specifically salt, cold, cadmium ion and osmotic stress. Among these abiotic stimuli, response to salt stress exhibited the highest degree of enrichment, indicating that graphene induced a kind of abiotic stress similar to salt stress. In contrast to up-regulated DEGs, the down-regulated DEGs were mainly enriched in ‘plant-type hypersensitive response’, ‘defense response’, ‘defense response to bacterium’, ‘defense response to biotic stimulus’, and ‘defense response to fungus (incompatible interaction)’, indicating that graphene treatment alleviated the plant biotic stress response. In addition, the response to external stimulus was accompanied by the alteration of biological processes related to the ‘response to hormone (abscisic acid)’, ‘signal transduction’, ‘gene expession’, ‘amino acid valine metabolism’, and ‘secondary metabolite synthesis (GDP-L-fucose and flavonoid)’. In addition, molecular function enrichment consisted of ‘copper ion binding’, ‘ADP binding’, ‘RNA-directed DNA polymerase activity’, ‘peroxidase activity’, ‘calcium ion binding’, ‘NAD(P)H oxidase activity’, and so on. Almost all the enriched molecular function terms were related to metabolism, suggesting that changes in metabolic activities play an important role in the plant’s response to graphene.

**Fig 5:**
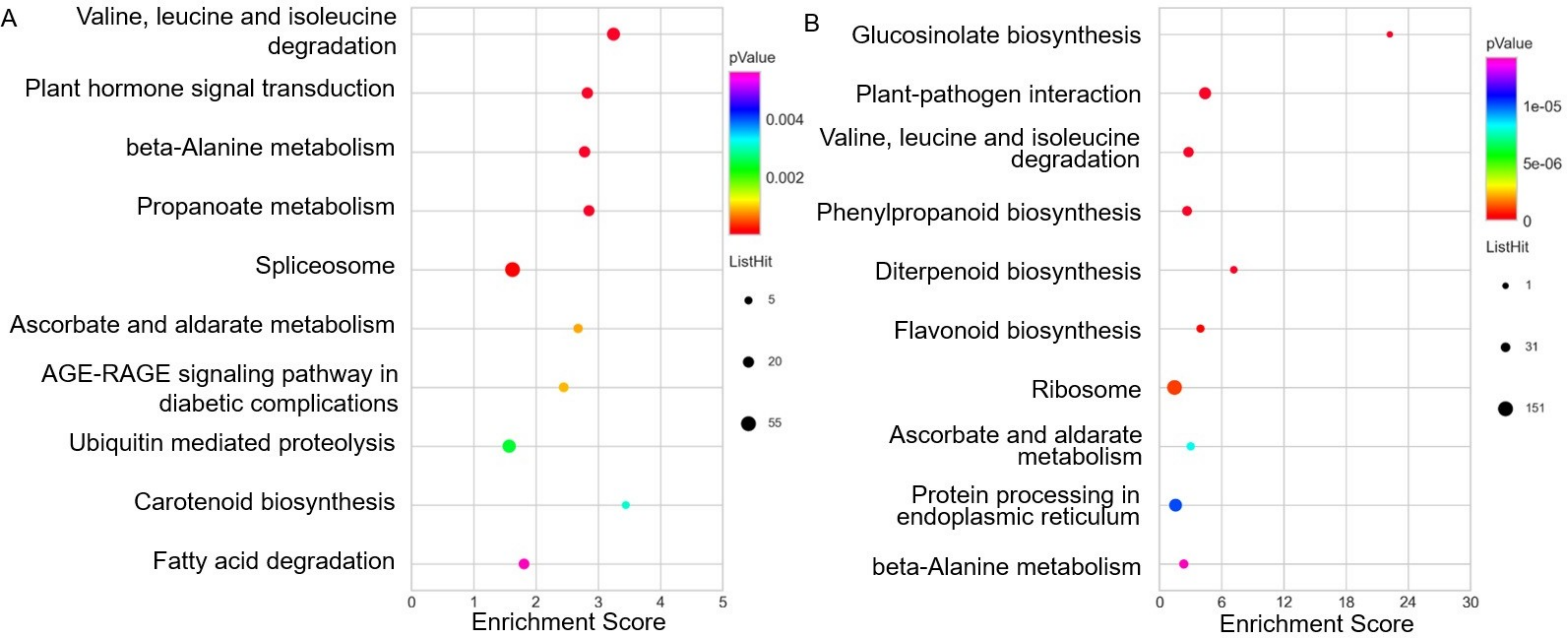
TOP10 KEGG enriched pathway based on DEGs annotated from plant databases. A: up-regulated DEGs; B: down-regulated DEGs.

To investigate the plant-related pathways affected by graphene, 1706 DEGs (694 up-regulated and 1012 down-regulated) annotated to plant database were also subjected to KEGG pathway analysis. Consistent with GO enrichment analysis, the DEGs up-regulated were enriched in categories related to plant hormone related signal transduction, while the DEGs down-regulated were enriched for the category ‘plant–pathogen interaction’ (Fig 5). The enriched KEGG pathways could be mainly divided into two classes: (1) environmental information processing, including plant–pathogen interaction and plant hormone signal transduction; (2) metabolism, involving the citrate cycle (TCA cycle), and the biosynthesis and degradation of many biomolecules, including amino acids, fatty acids, phenylpropanoids, flavonoids, propanoate, carotenoids, ascorbate and aldarate. In summary, KEGG analysis further indicates that graphene treatment results in alterations in hormone signal transduction and metabolism processes.

### Upregulated DEGs are enriched in plant response to abiotic stresses

Our observations using transmission electron microscopy to examine roots of graphene treated roots suggested the plants may be experiencing mechanical stress. This was further suggested by GO term enrichment analysis, which showed that up-regulated DEGs were enriched for terms related to responses to abiotic stress, especially salt stress. We further analyzed the DEGs related to responses to different abiotic stresses, including salt stress, water deprivation, cold, osmotic stress, cadmium ion and heat. Of the total of 6477 DEGs annotated to plant databases, there were 176 genes related to the salt stress response, including 99 up-regulated and 77 down-regulated, which made up the largest proportion of the different abiotic stresses (S3 Fig). In addition, the total number of DEGs related to responses to water deprivation, cold, osmotic stress, cadmium ion, and heat were 83, 89, 49, 94 and 74, respectively (S3 Fig).

**Fig 6:**
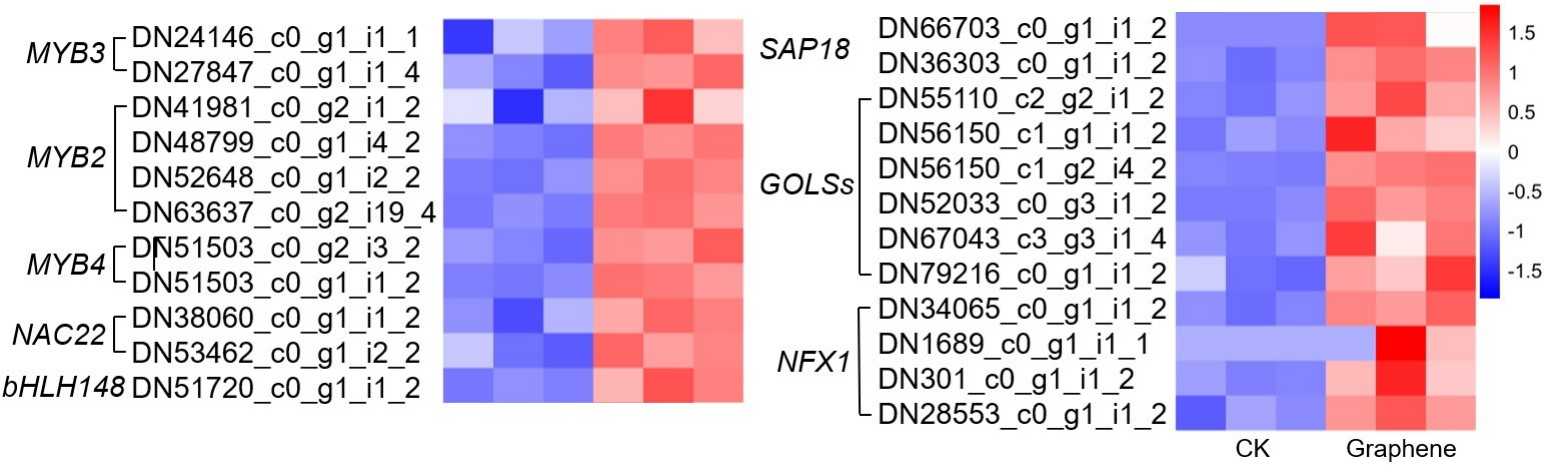
Selected candidate DEGs response to stress.

Many transcription factors are thought to be closely related to plant stress response, such as MYB family, NAC family, bZIP family, bHLH family, Zinc finger family, AP2/ERF family, and so on [37]. In addition, *SAP18* [38], *GOLS* [39] and *NFX1* [40] were reported to be induced by salt treatment. In order to clarify the effect of graphene treatment on plant salt stress response, we further analyzed the salt stress-related genes. As shown in Fig 6, the expression of *MYB3*, *MYB2*, *MYB4*, *NAC22*, *bHLH148*, *SAP18* (*Sin3A Associated Protein 18*), *GOLS* (*Galactinol synthase*) and *NFX1* (*NF-X-LIKE 1*) are up-regulated after six months of graphene treatment. *MYB3* was function in regulating anthocyanin biosynthesis and flower development in apple [41]. *MYB2* was involved in the patterning of proanthocyandin and anthocyanin pigmentation in *Medicago truncatula* [42]. Arabidopsis *MYB4* plays dual roles in flavonoid biosynthesis [43]. Rice *NAC22*, which down-regulated by virus infection, might be related to the health stage maintenance [44]. Overexpression of *OsbHLH148*, which induced by dehydration, high salinity, low temperature and wounding, leads to elevated tolerance to drought stress in rice [45]. *SAP18*, encoding a histone deacetylase complex subunit, was function in transcription regulation [38]. Overexpression of *TsGOLS2*, a galactinol synthase, conferred enhanced tolerance to high salinity and osmotic stresses in arabidopsis [39]. And the *AtNFXL1* gene, encoding a NF-X1 type zinc finger protein was required for growth under salt stress [40]. These candidate salt-stress response DEGs after graphene treatment suggested that graphene might activate salt-stress response pathways *in vivo*.

Many studies revealed that plant hormone signaling pathways, including abscisic acid (ABA), ethylene (ET), auxin, gibberellins (GAs), cytokinins (CKs), brassinosteroids (BRs), jasmonic acid (JA), and salicylic acid (SA), play key roles in responding to various external stresses and regulating plant growth and development [46]. KEGG pathway analysis of the DEGs revealed enrichment of genes involved in plant hormone signal transduction, and GO enrichment analysis highlighted the response to ABA. Therefore, we identified DEGs related to hormone signal transduction pathways and shown in S4 Fig. Among all the hormones, ABA and ET are most closely related to plant abiotic stress [47]. After graphene treatment, DEGs related to ABA and ET-related signaling pathways are enriched. We found 62 and 72 DEGs related to ABA and ET activated signaling pathways, respectively. The enrichment of DEGs in ABA and ET activated signaling pathways provide further evidence that graphene treatment induced stress responses in roots.

**Fig 7:**
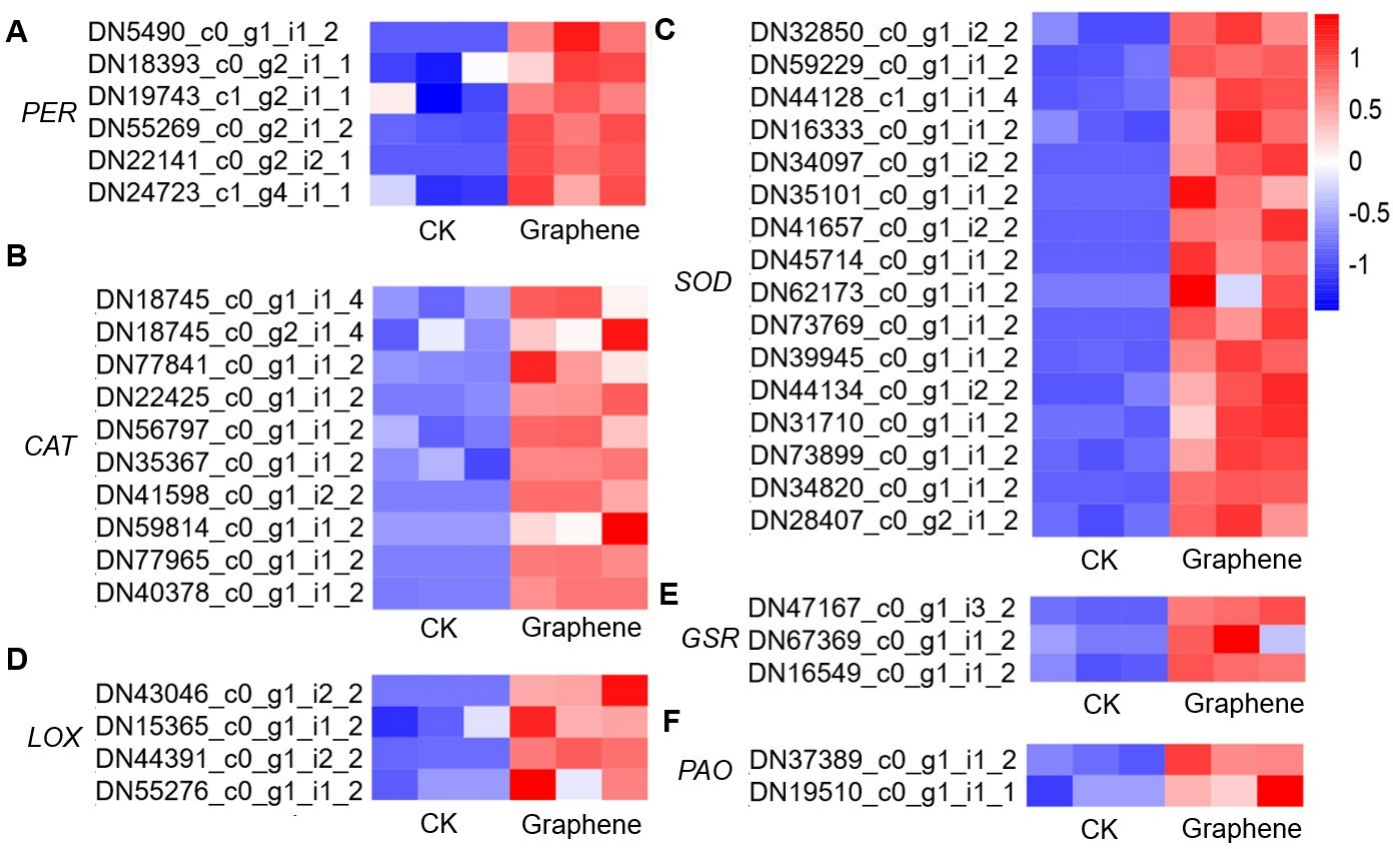
Selected metabolism related DEGs in response to stress.

Plants subjected to exogenous stress can have altered metabolic activities [48]. Many metabolism-related genes have been identified as markers of plant response to salt stress [49]. Under stress, the activity of enzymes that produce and scavenge ROS is affected, which can lead to changes in cellular ROS levels. ROS producers include lipoxygenase and polyamine oxidase, which were encoded by *LOX*, and *PAO*, respectively. And ROS scavengers include peroxidase, catalase and superoxide dismutase encoded by *PERs*, *CATs*, and *SODs*, respectively [50]. And glutathione producer glutathione reductase, encoded by *GR*, was critical to resisting oxidative stress [50]. There were 5 *PER*s, 8 *CAT*s, 20 *SOD*s, 3 *GRs*, 3 *LOXs*, and 3 *PAOs* genes up-regulated after graphene treated (Fig 7). These ROS-related genes can be used as representatives of stress-related metabolic pathways, and their up-regulated expression suggests that graphene treatment induced an *in vivo* stress response.

### DEGs downregulated were enriched in plant response to biotic stress

According to GO enrichment analysis and KEGG analysis, the down-regulated DEGs were enriched for the term ‘response to pathogen’. After graphene treatment, a total of 2120 predicted resistance genes (R-genes), based on PRGdb (http://www.prgdb.org), were differentially expressed [51], among which 795 were upregulated and 1325 were downregulated (S5 Fig). Strikingly, 4 *PR1* (*Pathogenesis- Related protein 1*) homologous genes were down-regulated. The down-regulated expression of a large number of R genes suggests that graphene treatment might lead to a decrease in plant immune activity. The hormones SA and JA are recognized as being important for plant immunity. After graphene treatment, the number of upregulated and downregulated DEGs related to SA and JA activated signaling pathway were almost equal (S3 Fig), which indicates that the observed down-regulation of R genes upon graphene treatment may be independent of SA and JA.

### DEGs regulated by graphene are involved in moderate stress promoting plant root growth

**Fig 8:**
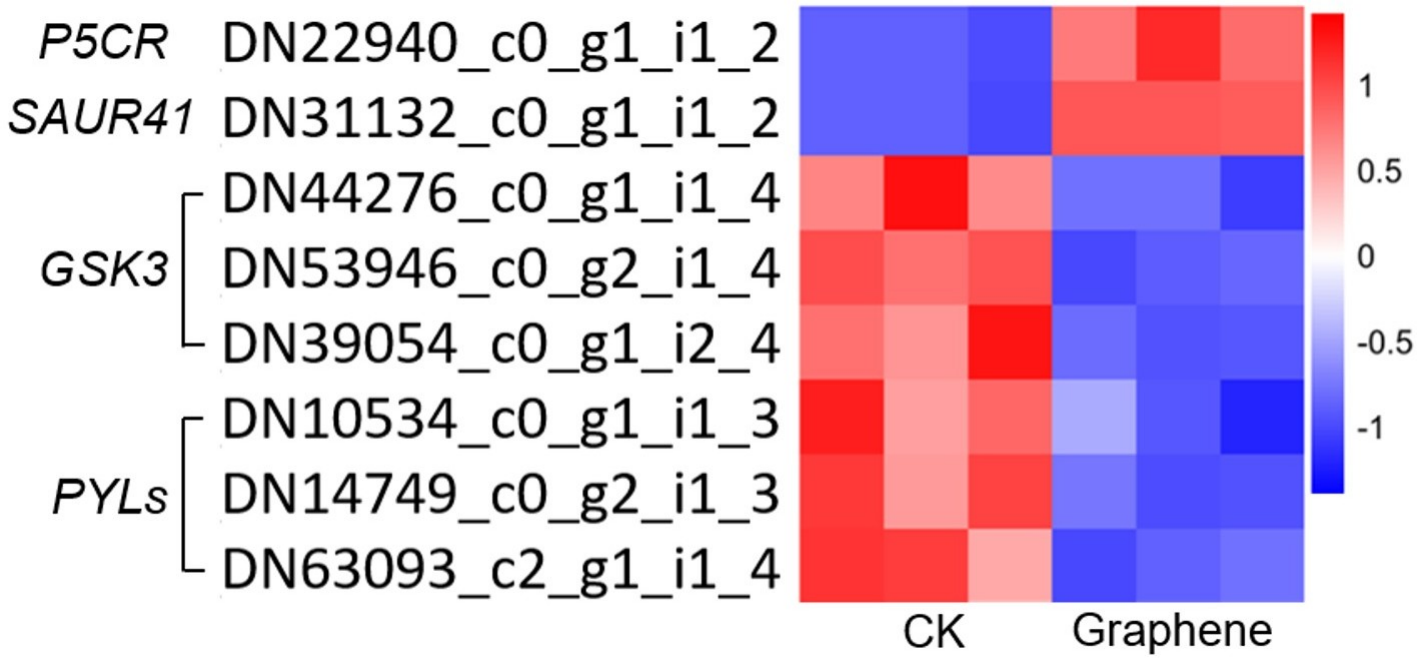
Selected DEGs promote root growth in response to stress.

Proline, the accumulation of which correlates with tolerance to drought and salt stress in plants, has been shown to affect root growth by controlling cell division [52, 53]. The *PYRROLINE-5-CARBOXYLATE REDUCTASE* (*P5CR)* gene, encoding pyrroline-5- carboxylate (P5C) reductase, was up-regulated after graphene treatment (Fig 8), which may trigger the increase of proline content and activate cell division. Interestingly, 62% (44) of the total 71 DEGs assigned to ‘cell cycle control’ and ‘cell division’ categories based on KOG classification, were upregulated, which further supports the promotion of cell division in graphene treated plants. In Arabidopsis, the *GLYCOGEN SYNTHASE KINASE 3* (*GSK3*) genes *ARABIDOPSIS THALIANA SHAGGY-RELATED KINASE 11* (*AtSK11*) and *AtSK12* are involved in the root growth response that occurs upon mild osmotic stress [54]. As shown in Fig 8, there are three GSK3 homologous genes downregulated, which could trigger root growth under mild osmotic stress. The *SMALL AUXIN UP-REGULATED RNAs* (*SAURs*) auxin-responsive genes are involved in the regulation of adaptive growth under abiotic stress [55]. There are 16 *SAURs* differently expressed after graphene treatment. *SAUR41*, which is considered as a positive regulator for cell and hypocotyl elongation, was upregulated by graphene [56]. Under abiotic stresses, such as salt stress, the PYLs (PYR/PYL/RCAR) ABA receptors are involved in root growth adaptation [57]. Mutations in *PYLs* promote plant growth and productivity [58]. Graphene treatment induced downregulation of all 3 *PYLs* (*PYL2*, *PYL3* and *PYL4*). The changed expression of these stress-responsive genes may therefore underlie the observed promotion of root growth in response to graphene treatments.

### Data validation

**Fig 9:**
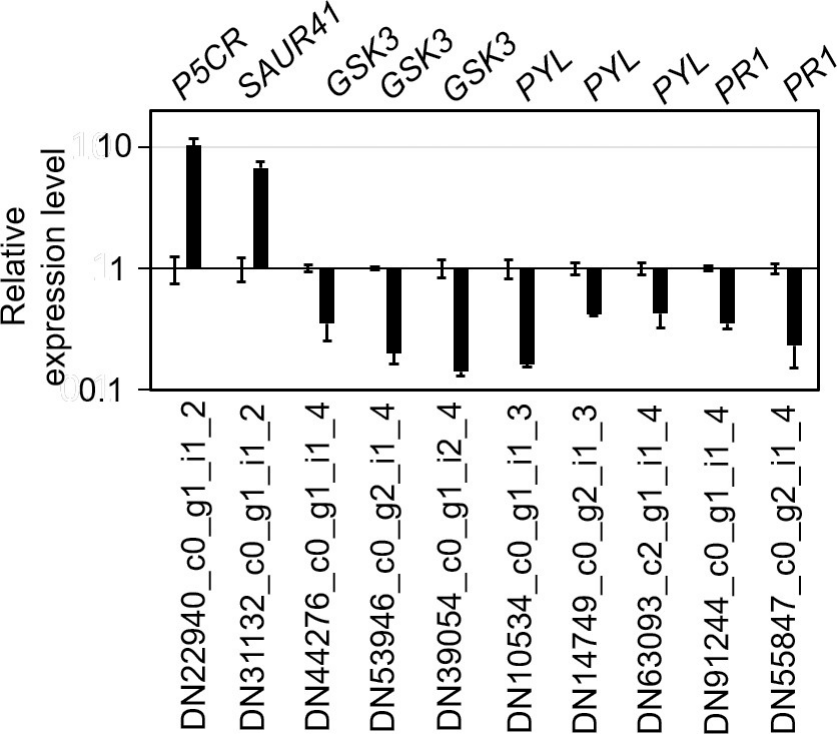
qRT-PCR validation of the RNA-seq results. Control group was set as 1, and the black column represented the relative expression of DEGs in the graphene-treated group.

The expression of 10 DEGs, including two *PR1* genes, *P5CR*, *SAUR41*, three GSK3 homologous genes and three *PYLs* were examined by real time PCR, to validate the RNA-seq results. The observed variation in expression of these genes in response to graphene treatment was consistent with that of transcriptome sequencing (Fig 9), indicating that the transcriptome sequencing results are reliable.

Our current understanding of the influence of graphene on woody plant growth is very limited. As a perennial woody plant, the sensitivity of *P*. *tabulaeformis* to exogenous substances may be different from that of herbs. Our study of *P*. *tabulaeformis* established that at a concentration of 25 mg/L, graphene addition to soil can stimulate plant growth, which not only provides evidence for the biological effect of graphene, but also provides a potential means to enhance forest productivity.

Physiological experiments showed that the effect of graphene on roots was stronger than on the aboveground tissues, which is consistent with research on *Aloe vera* [14]. This leads us to believe that the root system, which interacts with graphene directly, may be the most important organ in the plant’s response to graphene for both herbs and woody plants. Considering the correlation between root and aboveground growth, further studies are necessary to explore the effects of graphene on aboveground biomass by increasing the treatment time or changing the treatment conditions.

A large number of studies have shown that graphene treatment causes stress responses in plants that include the induction of oxidative stress [21, 59]. Our transmission electron microscope observations confirm that graphene treatment causes cell membrane damage in root cells. According to the results of GO enrichment analysis and KEGG pathway analysis, upregulated DEGs were enriched in genes related to salt stress. Graphene was reported to accelerate the absorption of heavy metals such as cadmium and arsenic in plants and to enhance the toxicity of heavy metals [20, 34, 60]. Mild salt stress may be induced in saline-alkali land, due to the increased salt uptake capacity of graphene treated roots.

For plants growing under natural conditions, moderate stress promotes the accumulation of proline [61], which greatly improves their ability to survive in the face of severe stress [52, 62]. The observed graphene induced expression of *P5CR* could affect proline content. In addition, many hormones play important roles in mediation of stress responses and regulation of plant growth adaptation [63]. Among these, ABA is the most important for abiotic stress responses. GO analysis showed that DEGs related to the ABA response were enriched in graphene treated roots. In addition, negative root growth regulators *PYL* ABA receptors [58] and *GSK3* homologous genes [54] were down-regulated by graphene treatment, while positive root growth regulators *SAUR* auxin response genes [55, 56] were up-regulated by graphene treatment. These genes may be involved in graphene-induced plant growth and are attractive candidate genes for further research.

Graphene has an antibacterial effect on both bacterial and fungal pathogens, and can significantly change the structure of the soil microbial community [16, 17]. The observed effects of graphene on the plant biotic response may be caused by suppression of microbial populations. Graphene induced the down-regulation of a large number of resistance genes, but did not significantly disturb the JA and SA signaling pathways. Defense responses are always costly and can lead to the inhibition of growth [64], therefore the observed decrease in defense responses may also be an important factor in graphene-induced plant growth promotion.

Due to their sessile nature, plants are always exposed to an ever-changing environment throughout their growth cycle and need to constantly adjust to the trade-off between growth and defense [65]. The mild stress response induced by graphene may prime plants, making them more resistant to stress, while graphene’s strong antimicrobial ability may help plants conserve energy that would otherwise be needed for dealing with pathogens. Our study brings a new perspective on the biological effects of graphene and provides a theoretical basis for the application of graphene in agriculture and forestry.

## Conclusions

The treatment of bare-rooted seedlings of *P*. *tabulaeformis* with graphene promoted root growth with the highest efficiency at 25 mg/L, but damaged the cell membranes of root cells. Graphene triggered abiotic stress responses but depressed the biotic stress response. In addition, metabolic processes and hormone signal transduction pathways were altered in graphene-treated plants. The growth promoting effects of graphene may be mediated by increased proline synthesis, and the reduced expression of the *SAUR41* auxin response gene, ABA receptor encoding *PYLs* genes, and *GSK3* homologous genes, which are attractive candidate genes for future research.

## Supporting information

S1 Fig Representative photographs of *P*. *tabulaeformis* plants after treatment with graphene for 6 months. (TIF)

S2 Fig GO functional classification of total unigenes. (TIF)

S3 Fig DEGs response to different abiotic stress stimuli. (TIF)

S4 Fig DEGs involved in hormone mediated signaling pathways. (TIF)

S5 Fig DEGs of resistance genes. (TIF)

S1 Table Clean data of 6477 plant DEGs. (XLSX)

S1 File Result and methods for total gene functional annotation and classification. (DOCX)
