# Supplementary figures and images for: Effects of graphene on morphology, microstructure and transcriptomic profiling of Pinus tabuliformis Carr. roots
Source: PLoS One. 2021 Jul 8;16(7):e0253812. doi: 10.1371/journal.pone.0253812 (PMC8266090; doi:10.1371/journal.pone.0253812)

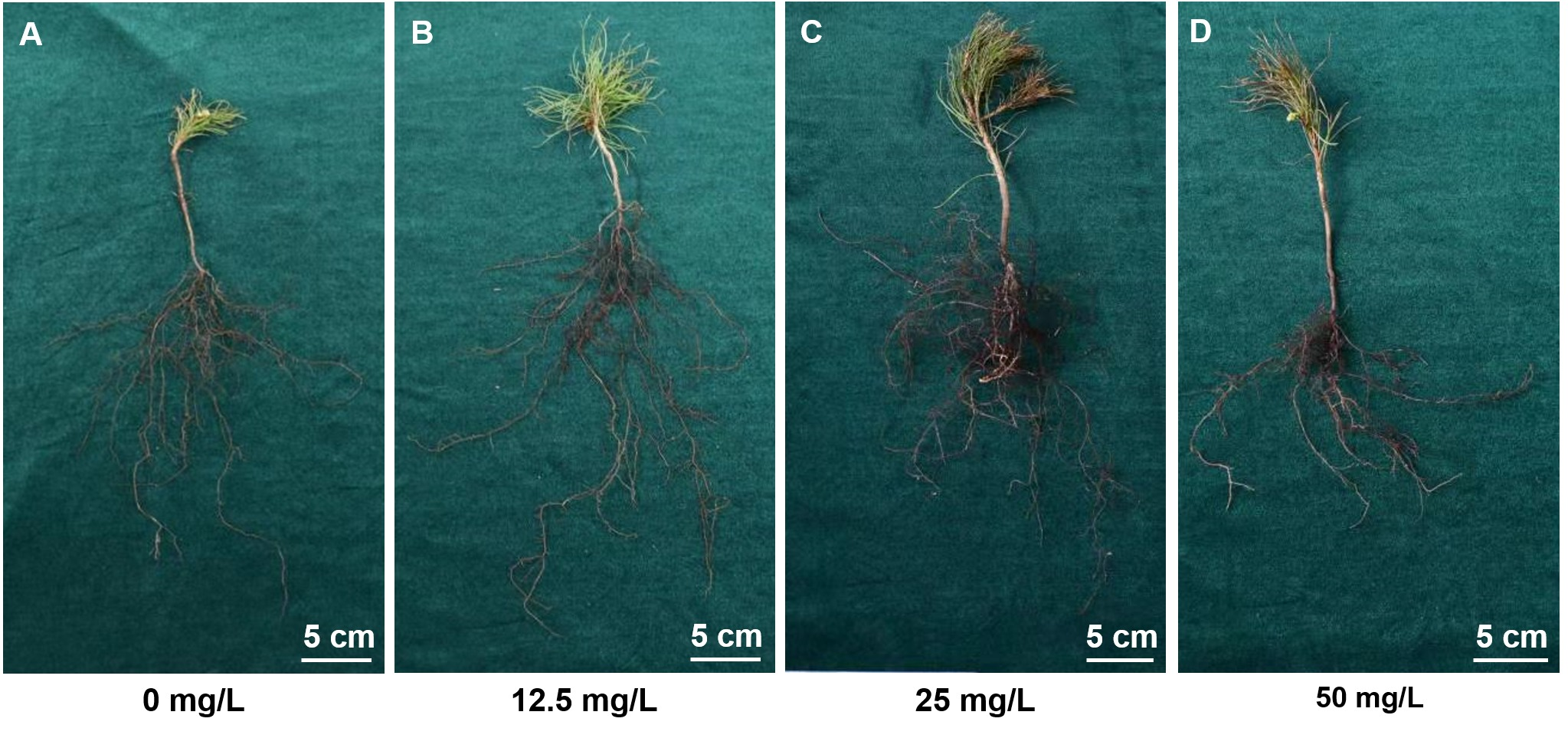

Supplement: S1 Fig — (TIF) [file pone.0253812.s001.tif]

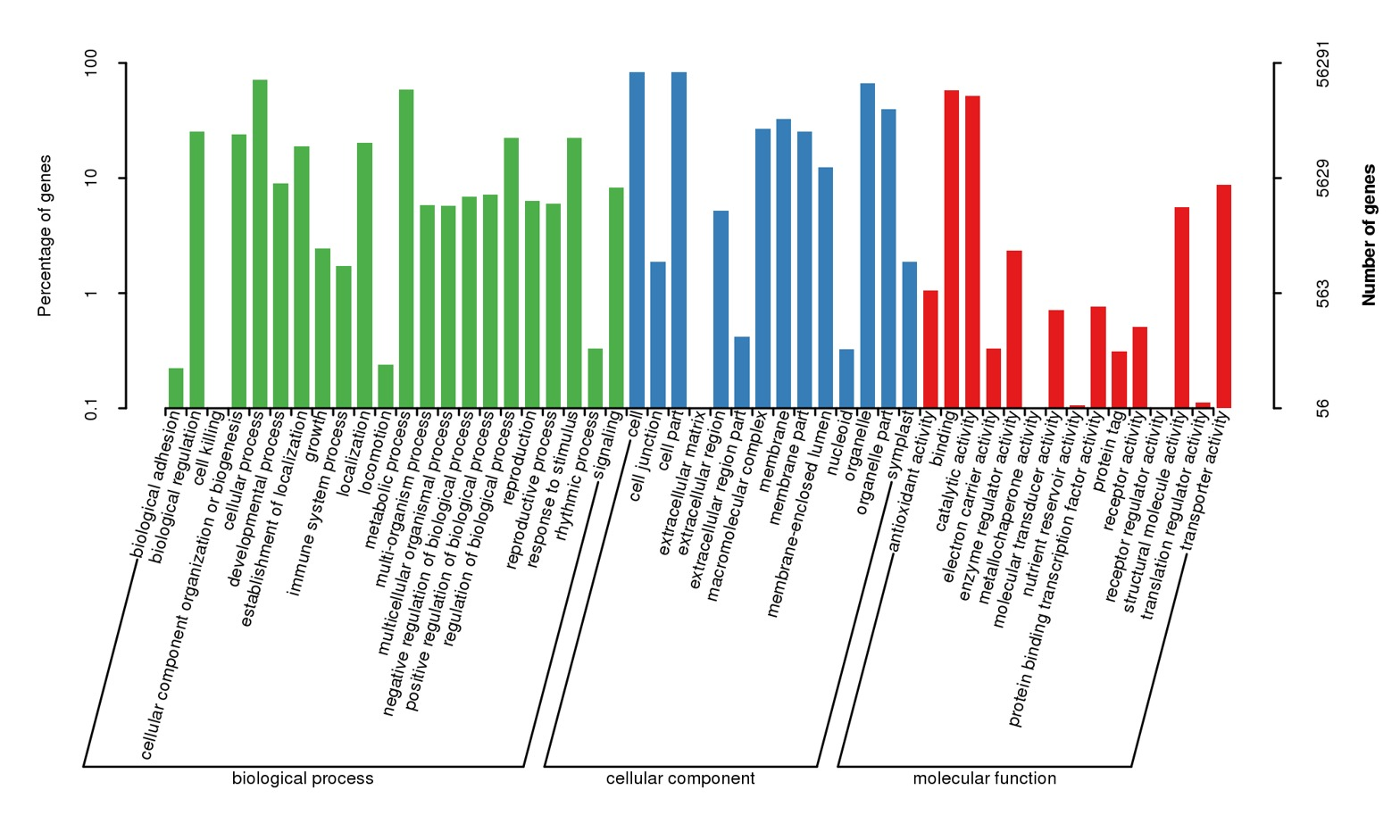

Supplement: S2 Fig — (TIF) [file pone.0253812.s002.tif]

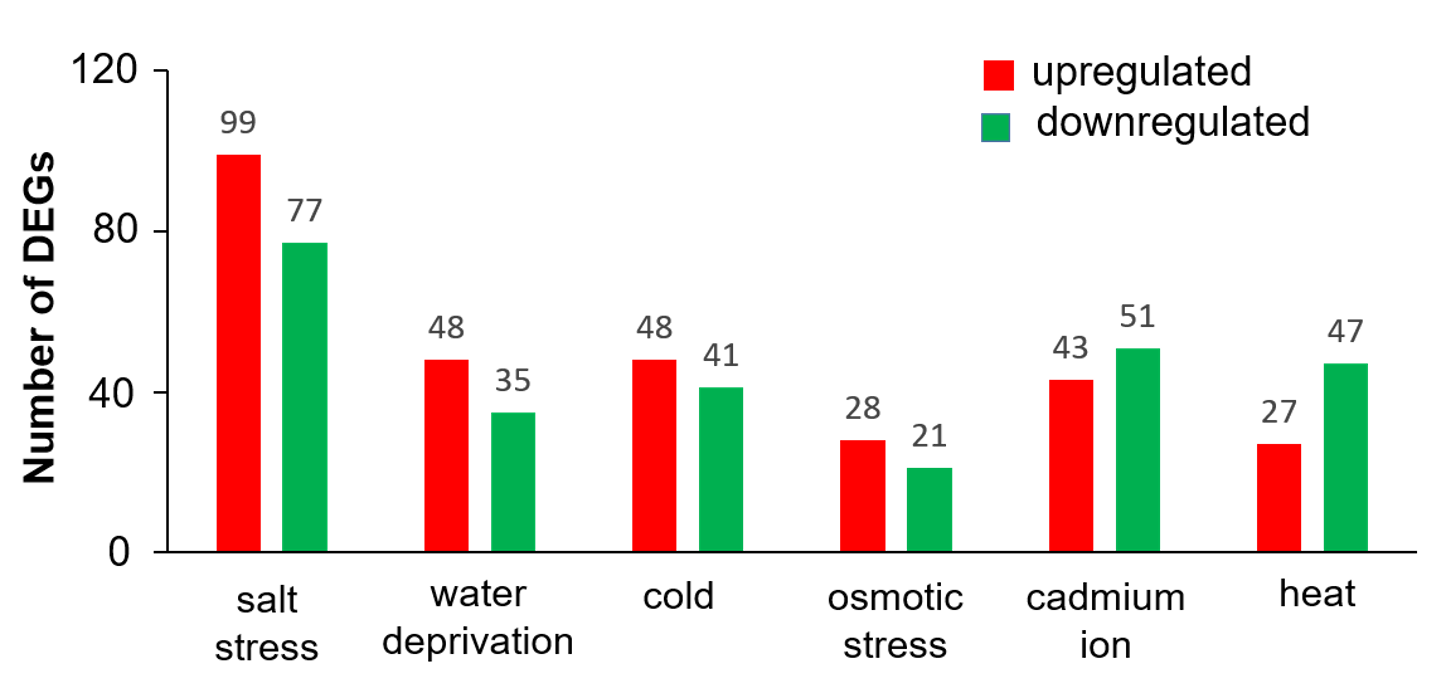

Supplement: S3 Fig — (TIF) [file pone.0253812.s003.tif]

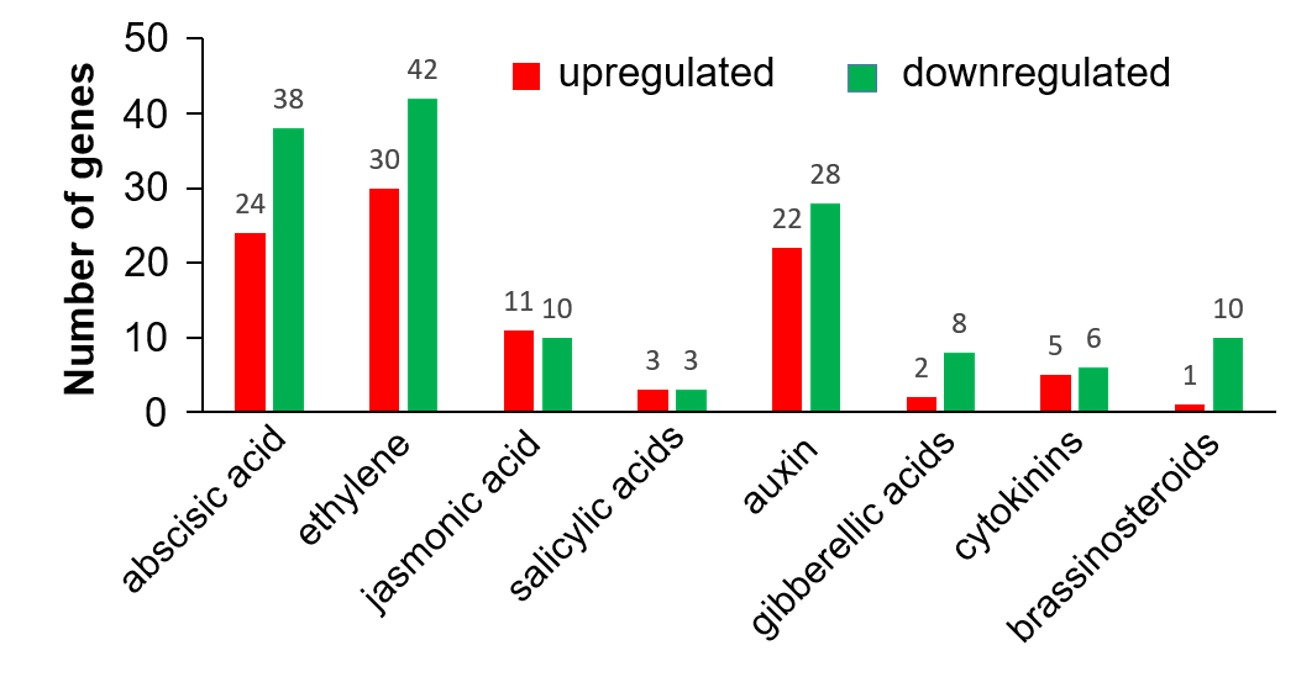

Supplement: S4 Fig — (TIF) [file pone.0253812.s004.tif]

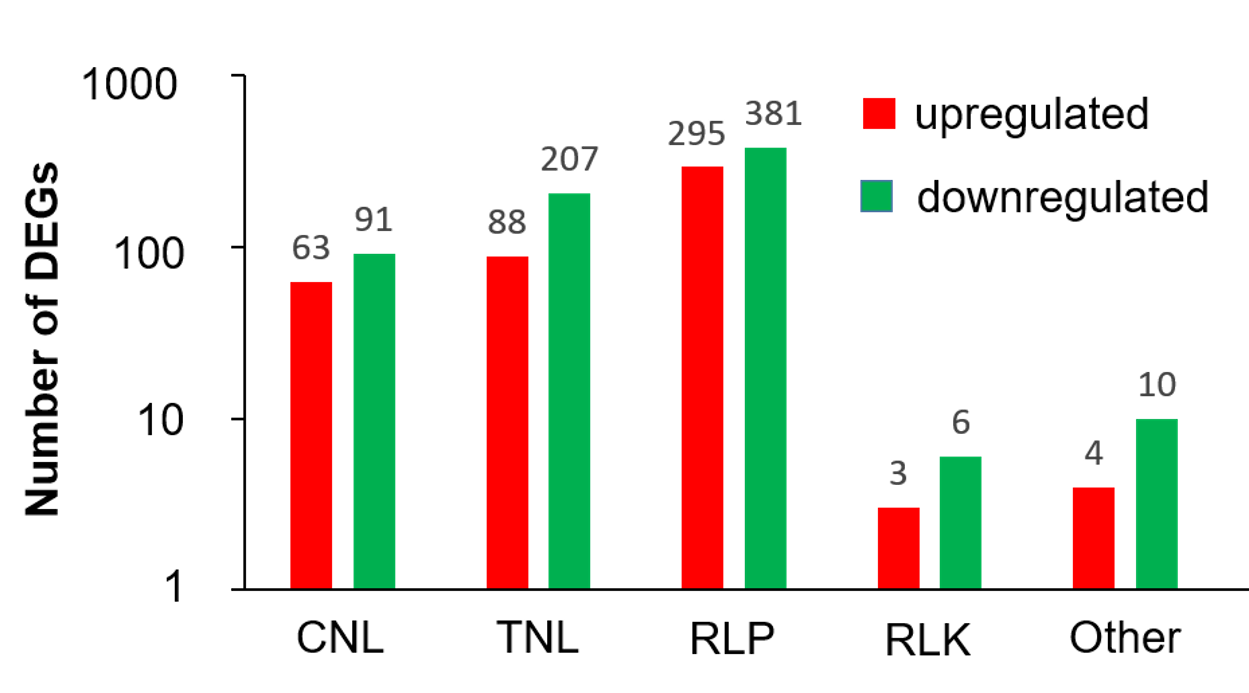

Supplement: S5 Fig — (TIF) [file pone.0253812.s005.tif]
