# Supplementary material for: Effects of graphene on morphology, microstructure and transcriptomic profiling of Pinus tabuliformis Carr. roots
Source: PLoS One. 2021 Jul 8;16(7):e0253812. doi: 10.1371/journal.pone.0253812 (PMC8266090; doi:10.1371/journal.pone.0253812)
Supplement: S1 File — (DOCX) [file pone.0253812.s007.docx]

**S1 File.**

**Functional annotation and classification**

All assembled unigenes were functionally annotated by searching against NR, KOG, GO, SWISS-PROT, EggNOG, and KEGG databases, with an E-value threshold of 1E^-5^. The number of unigenes matched to NR, KOG, GO, SWISS-PROT, EggNOG, KEGG and Pfam databases were 94405 (76.5%), 48072 (38.9%), 56291 (45.6%), 60182 (48.8%), 81013 (65.6%), 35719 (28.9%) and 59351 (48.1%). In the NR database, the unigenes matched sequences mainly from the genome of *Picea sitchensis* (14.0%), *Tuber borchii* (10.4%), *Suillus luteus UH-Slu-Lm8-n1* (9.7%), *Pyronema omphalodes CBS 100304* (9.1%), *Neonectria ditissima* (4.3%), *Rhizopogon vinicolor AM-OR11-026* (4.2%), *Rhizopogon vesiculosus* (3.6%), *T. aestivum* (3.3%), *T. magnatum* (2.9%) and *Amborella trichopoda* (2.5%). It is worth noting that *T. borchii, T. aestivum, T. magnatum, Rhizopogon vinicolor,* and *Suillus luteus* are **ectomycorrhizal** symbiotic **fungi** that colonize roots of *Pinus* or other evergreen species, suggesting that the sequences are of fungal origin and that the plants studied were colonized by these fungi.

Based on Swissprot database, the possible fuctions of the unigenes were classified using Gene Onthology classification system, which consist of three main categories (biological processes, cellular components, and molecular function). The two terms with the highest number of genes in biological processes category were cellular process (40120, 71.27%) and metabolic process (33099, 58.80%). Cell (47246, 83.93%) and cell part (47164, 83.79%) were the most abundant groups in cellular component category. Within the molecular function category, binding (32552, 57.83%) and catalytic activity (29158, 51.80%) were the most abundant groups (Fig S2).

**Supplemental Methods:**

The functional annotation of the unigenes was carried out through their alignment with the Non-redundant (NR) database (http://www.ncbi.nlm.nih.gov/), SSWISS-PROT (http://www.expasy.ch/sprot/), and Clusters of orthologous groups for Eukaryotic Orthologous Groups of proteins (KOG) database (http://www.ncbi.nlm.nih.gov/cog/) using BLASTX with a threshold E-value of 10^-5^. The proteins with the highest hits to the unigenes were used to assign functional annotations.


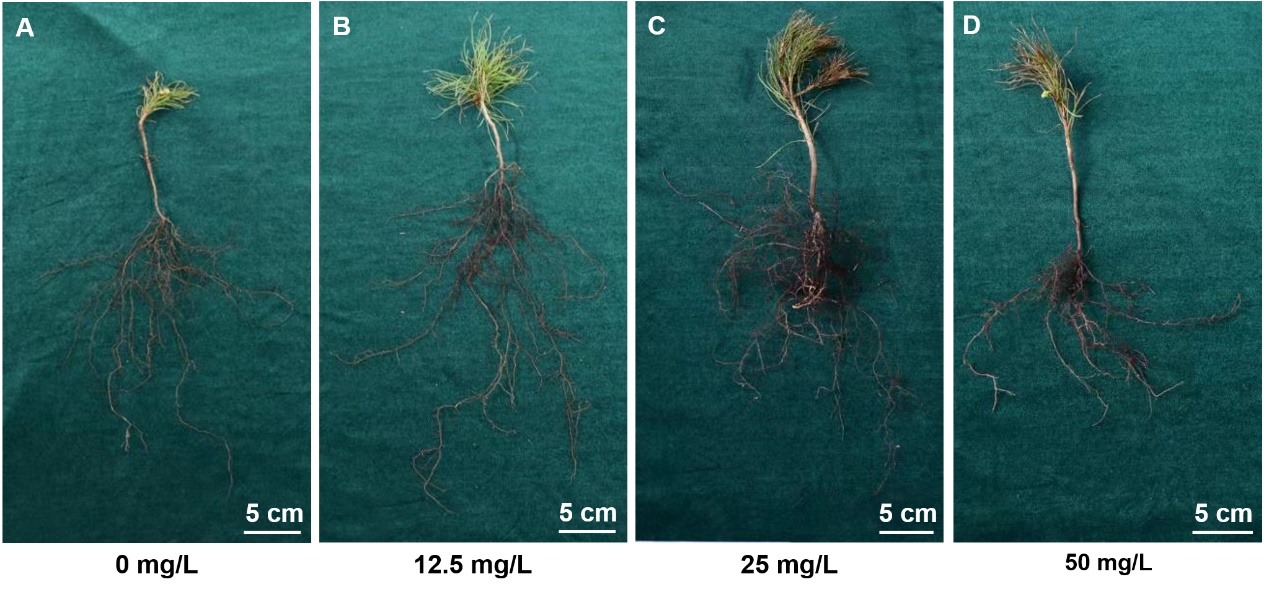


**Figure S1 Representative photographs of *Pinus tabulaeformis* plant after treatment with graphene for 6 months.**

| TRINITY_DN15602_c0_g1_i1_1-F: | AAGTTGGTCTGTGCCAGATG |
| --- | --- |
| TRINITY_DN15602_c0_g1_i1_1-R: | CACTAAACAGAGCAGCCACT |
| TRINITY_DN22940_c0_g1_i1_2-F: | GCGTACTAGGATGTGGAACG |
| TRINITY_DN22940_c0_g1_i1_2-R: | GTTGCAGTGAAAATGGACGG |
| TRINITY_DN31132_c0_g1_i1_2-F: | CCATTTATGTGGGGAGCGAG |
| TRINITY_DN31132_c0_g1_i1_2-R: | TCCAATTTTCCGGCAGACAG |
| TRINITY_DN44276_c0_g1_i1_4-F: | ACCAAGATTGACGTGTGGTC |
| TRINITY_DN44276_c0_g1_i1_4-R: | CTGTTCTCGAGTAGGCGTTC |
| TRINITY_DN53946_c0_g2_i1_4-F: | TTGACGTAGGTCGGGTTTTG |
| TRINITY_DN53946_c0_g2_i1_4-R: | GGACGAAGTAAGGAACCGAC |
| TRINITY_DN39054_c0_g1_i2_4-F: | AGCTAGCTCAGATGCGATTG |
| TRINITY_DN39054_c0_g1_i2_4-R: | AGAATGGATGGCACATTGCT |
| TRINITY_DN10534_c0_g1_i1_3-F: | GAGATTCGACCAGCCTCAAG |
| TRINITY_DN10534_c0_g1_i1_3-R: | GGAAGACCCGAAACAACACT |
| TRINITY_DN14749_c0_g2_i1_3-F: | ATCCCGTTTGGTCTGAGAGA |
| TRINITY_DN14749_c0_g2_i1_3-R: | AGCTCCGTATGAAGTGCTTG |
| TRINITY_DN63093_c2_g1_i1_4-F: | CCATCACCAGTCTTCACGAG |
| TRINITY_DN63093_c2_g1_i1_4-R: | CTTTCGTGTTTCCATGTGGC |
| TRINITY_DN91244_c0_g1_i1_4-F: | GAAGGCGTATGGTGTACCTG |
| TRINITY_DN91244_c0_g1_i1_4-R: | AAACCGCTGGTTTAGGACTC |
| TRINITY_DN55847_c0_g2_i1_4-F: | TGCTCAAGATTGGGCTGAAC |
| TRINITY_DN55847_c0_g2_i1_4-R: | ATGTGCCGGTATTAGAGCAG |

**Table S1 PCR primers used for qRT-PCR in this study**

| **Title 1** | **FW (g)** | **DW (g)** | **WC (%)** |
| --- | --- | --- | --- |
| 0 | 1.32±0.20c | 0.48±0.09b | 63.71±2.88 |
| 12.5 | 2.21±0.20b | 0.80±0.06a | 63.88±2.72 |
| 25 | 2.77±0.64a | 0.98±0.20a | 64.46±1.73 |
| 50 | 2.57±0.62ab | 0.96±0.28a | 63.00±3.04 |

**Table S2: Root biomass of *Pinus tabulaeformis* seedling after different concentrations of graphene treatment.**

Note: FW, Fresh weight; DW, Dry weight; WC, Water content.

| **C (mg/L)** | **RL (cm)** | **RP (cm^2^)** | **RS (cm^2^)** | **RV (cm^3^)** | **RT** | **RF** |
| --- | --- | --- | --- | --- | --- | --- |
| 0 | 559.54±58.38c | 29.47±3.27c | 92.57±10.29c | 1.22±0.18b | 1275.75±224.06b | 2899.75±486.34b |
| 12.5 | 755.82±54.56a | 45.78±2.81b | 143.81±8.84b | 2.19±0.25a | 1599.75±216.57a | 3480.63±502.67a |
| 25 | 814.49±83.82a | 49.51±5.23a | 155.53±16.43a | 2.37±0.34a | 1781.92±202.51a | 3620.9±339.24a |
| 50 | 650.00±40.49b | 42.09±3.18b | 132.22±9.98b | 2.16±0.35a | 1285.88±202.89b | 2957.25±191.84b |

**Table S3: Root morphology characters of *Pinus tabulaeformis* seedling after different concentrations of graphene treatment.**

Note: C, concentration of graphene; RL, Root length; RP, Root projected area; RS, Root superficial area; RV, Root volume; RT, Root tip number; RF, Root fork number.

| **Sample** | **Raw Reads** | **Clean Reads** | **Percent of valid bases** | **GC%** | **Total mapped reads** | **Uniquely mapped** | **Multiple mapped** |
| --- | --- | --- | --- | --- | --- | --- | --- |
| CK-1 | 51.96 M | 51.47 M | 96.41% | 46.41% | 44.37 M | 35.55 M | 8.82 M |
| CK-2 | 55.56 M | 55.04 M | 96.46% | 46.36% | 47.55 M | 38.08 M | 9.47 M |
| CK-3 | 50.81 M | 50.34 M | 96.40% | 46.38% | 43.40 M | 34.72 M | 8.69 M |
| 25-1 | 52.97 M | 52.48 M | 94.87% | 46.51% | 46.70 M | 37.08 M | 9.62 M |
| 25-2 | 56.83 M | 56.27 M | 96.29% | 46.45% | 50.18 M | 40.06 M | 10.12 M |
| 25-3 | 51.78 M | 51.29 M | 96.32% | 46.48% | 45.73 M | 36.48 M | 9.26 M |

**Table S4: Statistical data of sequencing and assembling results**


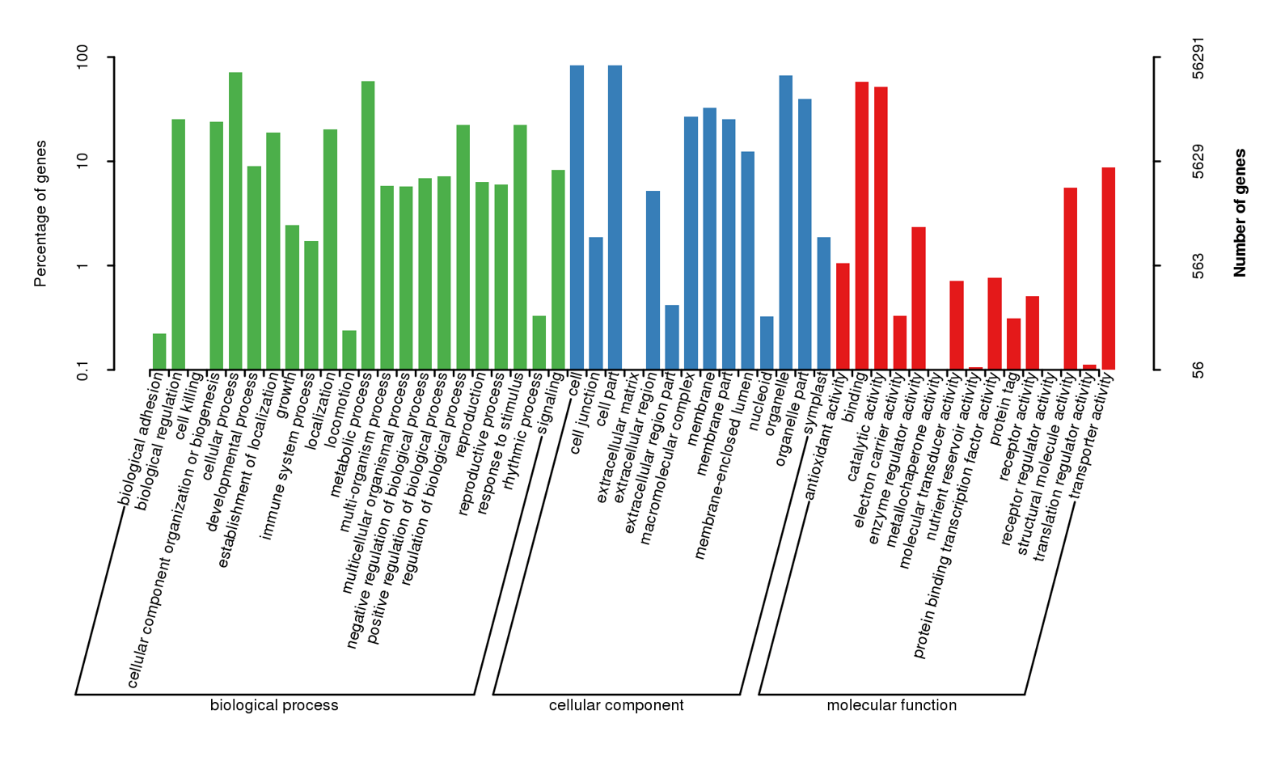


**Figure S2 GO functional classification of total unigenes**


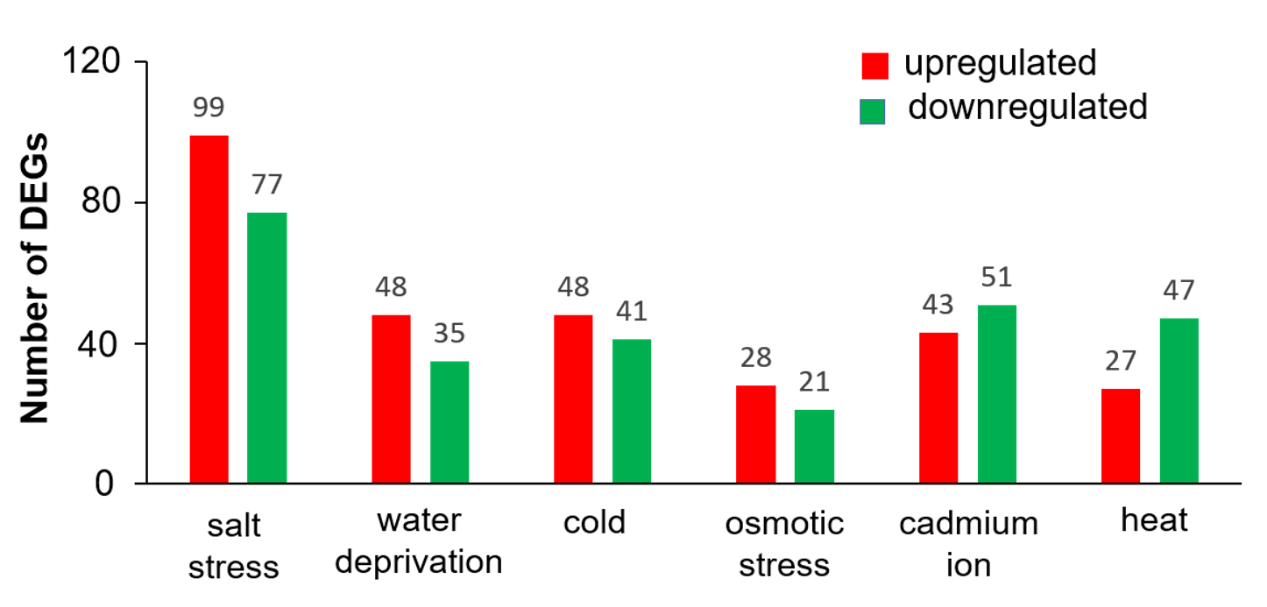


**Fig S3. DEGs response to different abiotic stresses stimulus**


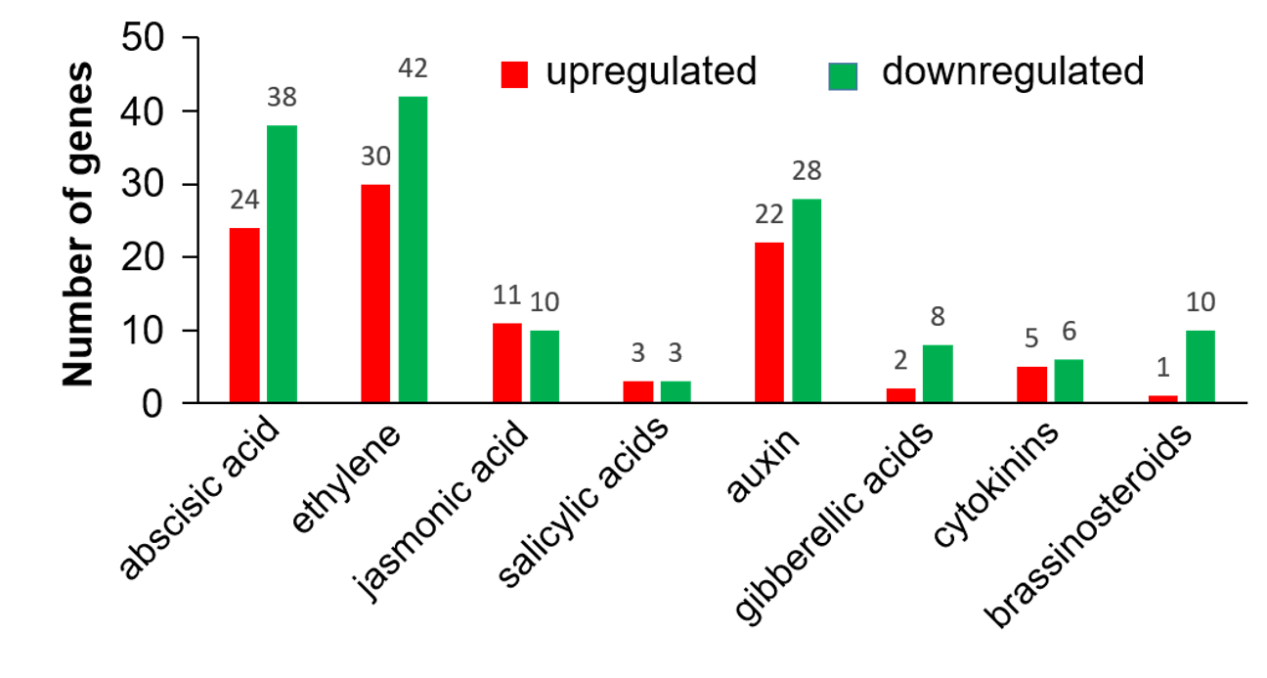


**Fig S4. DEGs involved in hormone mediated signaling pathway**

*

*

**Fig S5. DEGs of resistant genes**
